# Preparation of Ion-Exchanged TEMPO-Oxidized Celluloses as Flame Retardant Products

**DOI:** 10.3390/molecules24101947

**Published:** 2019-05-21

**Authors:** Cunzhen Geng, Zhihui Zhao, Zhixin Xue, Peilong Xu, Yanzhi Xia

**Affiliations:** 1 State Key Laboratory of Bio-fibers and Eco-textiles, Qingdao University, Qingdao 266071, China; 2 Institute of Marine Biobased Materials, Qingdao University, Qingdao 266071, China; zzh@qdu.edu.cn (Z.Z.); xuezhixin@qdu.edu.cn (Z.X.); 3 Co-Innovation Center for Marine Biomass Fibers, Materials and Textiles of Shandong Province, Qingdao University, Qingdao 266071, China

**Keywords:** cellulose, TEMPO-oxidized cellulose (TOC), thermal stabilization, microscale combustion calorimeter (MCC), flame retardancy

## Abstract

Cellulose, as one of the most abundant natural biopolymers, has been widely used in textile industry. However, owing to its drawbacks of flammability and ignitability, the large-scale commercial application of neat cellulose is limited. This study investigated some TEMPO-oxidized cellulose (TOC) which was prepared by selective TEMPO-mediated oxidation and ion exchange. The prepared TOC was characterized by Fourier transform infrared (FT-IR) spectroscopy and solid-state ^13^C-nuclear magnetic resonance (^13^C-NMR) spectroscopy. The thermal stability and combustion performance of TOC were investigated by thermogravimetry analysis (TG), microscale combustion calorimetry (MCC) and limiting oxygen index (LOI). The results demonstrated that the thermal stability of TOC was less than that of the pristine material cellulose, but the peak of heat release rate (pHHR) and the total heat release (THR) of all TOC were significantly reduced. Additionally, the LOI values of all TOC products were much higher 25%. In summary, the above results indicated that the modified cellulose with carboxyl groups and metal ions by selective oxidation and ion exchange endows efficient flame retardancy.

## 1. Introduction

Cellulose, the most abundant renewable polysaccharide on Earth, has safe, biocompatible, hydrophilic, and biodegradable natures, and is one of the best candidates for textile and other functional materials. Cellulose has broad potential in the design of advanced polymeric materials because of its linear (1,4)-β-glucan structure with three reactive hydroxyl groups per anhydroglucopyranose unit [1]. It is well-known that the cellulose is also one of the most flammable materials (limiting oxygen index (LOI) 18.4%) and can be ignited easily [2,3]. However, it is of primary importance for public safety to find ways to render this material less flammable and, of course, in a most economical and environment friendly manner. Consequently, to endow cotton fabric with excellent flame retardancy, flame-retardant modification is necessary.

In the last years, many flame retardants which contained halogen, phosphorous, nitrogen, sulfur, silicon, boron, and aluminum have been studied to endow cellulose with highly efficient flame-retardant property [4,5,6,7]. Although these flame retardants can effectively enhance the flame-retardancy of textile, some have been shown to be hazardous for human health and the ecological environment. Moreover, some of these flame retardants are easily washed out and not durable. Therefore, it is necessary to develop other methods to improve the flame retardancy of cellulose, for instance, some stable groups (anionic phosphate groups) are introduced to cellulose macromolecular chain through chemical bond to improve the flame-retardant property of cellulose [8].

Alginic acid, a kind of linear polysaccharide, is derived from brown seaweed. It is a co-polymer composed of b -1, 4-d-mannuronate (M) and a -1,4-l–guluronate (G) repeating monomeric units with the chemical formula (C_6_H_8_O_6_)_n_. In our laboratory, we already found that alginate fibers have a potential and intrinsically flame-retardant property without the addition of any other flame retardants. Kong, Q.S. et al. found that calcium alginate fibers, prepared by wet spinning of sodium alginate into a coagulating bath containing calcium chloride, are inherently flame retardant with a LOI value of 34% [9]. Zhang, J.J. et al. did further research, which demonstrated that calcium alginate fiber was intrinsically flame retardant with LOI value of 48% and proposed a condensed phase mechanism for the calcium alginate fiber flame retardancy effect [10]. Alginate fibers usually contain some Na, Ca, or other salts, and the presence of these ions will increase their flame-retardant property. More importantly, the presence of a carboxylate/carboxylic acid group will behave as a Lewis acid when alginate is heated, thereby producing char and increasing the value of LOI. The most typical method to introduce carboxyl groups into cellulose is carboxymethylation with monochloroacetic acid under alkaline conditions generally containing i-propanol, and large amounts of carboxymethyl cellulose (CMC) have been produced at industrial level.

**Figure 1:**
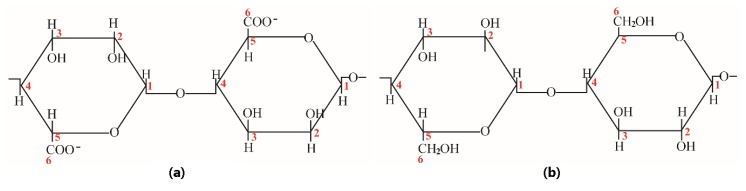
Chemical structure of alginate (**a**) and cellulose (**b**).

By comparing the two chemical structures of alginate and cellulose (Figure 1), a distinct difference is that a primary hydroxyl group located in C6 position of cellulose and a carboxylic acid group in corresponding position of alginate.

Based on this information, we tried to find a method to realize the transformation from a primary alcohol group to a carboxyl group in C6 position. A 2,2,6,6- tetramethylpiperidine-1-oxyl radical (TEMPO)—mediated oxidation is a system of the position—selective chemical modifications of primary hydroxyl groups, undertaken by reaction with TEMPO, sodium hypochlorite, and sodium bromide in water. It was an effective method to convert the primary hydroxyl groups of polysaccharides, such as sulfite pulp, cotton, bacterial cellulose, etc., to carboxyl groups with high selectivity [11,12,13,14].

In the present paper, we attempted to selectively oxidize the wood pulp whose chemical structure is the same as cellulose by the TEMPO-mediated oxidation system in water to obtain TEMPO-oxidized cellulose (TOC). Furthermore, the burning behavior and thermal stability of TOC were further investigated by MCC tests and TG analysis, respectively. To the best of our knowledge, this is the first reported flame-retardant behavior observed in cellulose by modifying the C6 group in its molecular structure.

## 2. Results and Discussion

### 2.1. FT-IR Spectroscopy Analysis

**Figure 2:**
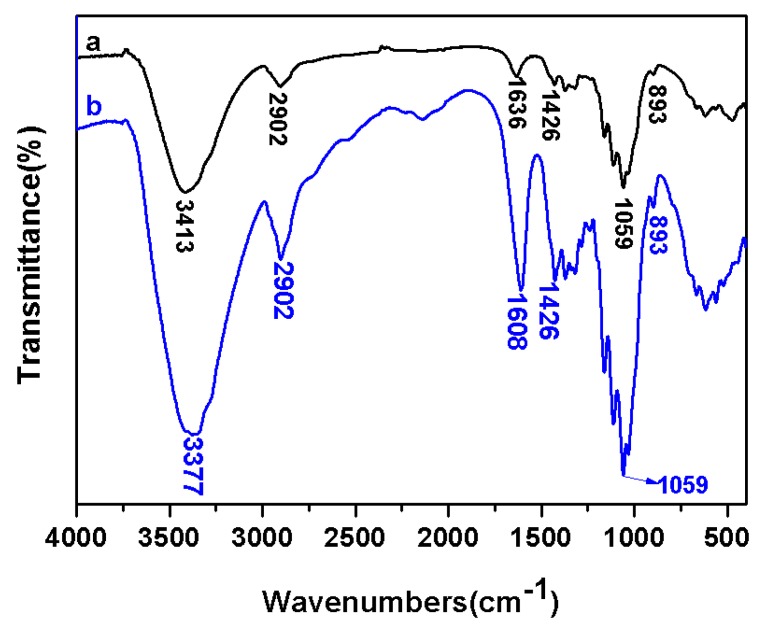
FT-IR spectra of the original cellulose (a) and cellulose-COONa (b).

FT-IR spectra of the cellulose and cellulose-COONa are shown in Figure 2. The wood pulp has almost no absorption band from 1400 to 1700 cm^−1^, but two strong absorption bands at 1608 and 1426 cm^−1^ derived from the carbonyl groups present in response to the TEMPO-mediated oxidation of wood pulp, which are used for quantification purposes representing the appearance of ν_as_(C=O) and ν_s_(C-O), respectively. In addition, a slight reduction of the bands related to hydrogen bonds ν(OH) at 3377 cm^−1^ and ν(CH) at 2902 cm^−1^ were observed. The results are consistent with other reports [14,15]. From these results, it can be concluded that hydroxyl groups at the C6 position of cellulose molecules are converted to sodium carboxylate and the wood pulp can be selectively oxidized in C6 position of cellulose molecules with TEMPO-mediated oxidation system.

**Figure 3:**
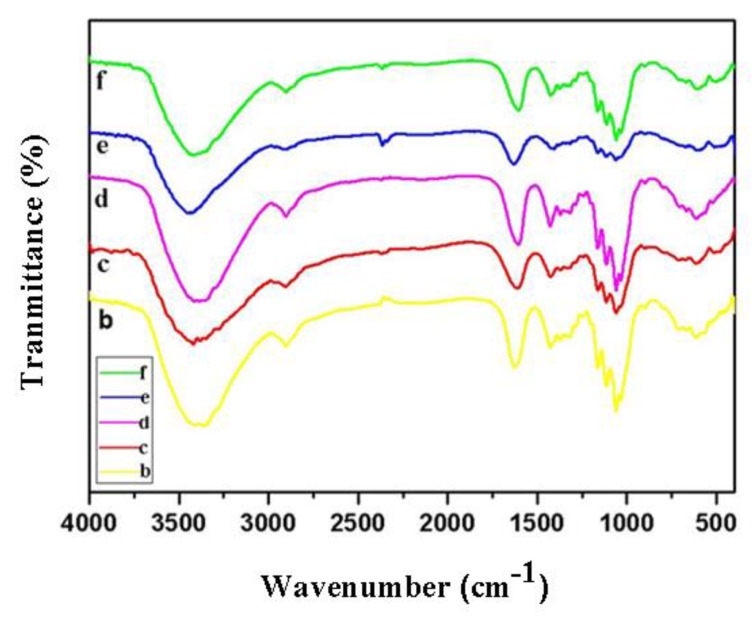
FT-IR spectra of cellulose-COOZn (b), cellulose-COOMg (c), cellulose-COOCa (d), cellulose-COONa (e), and cellulose-COOBa (f).

Additionally, all the FT-IR spectra of TOC are presented in Figure 3. The peak at 1608 cm^−1^ was attributed to the antisymmetric stretching vibration peak of carboxylic group (–COO- )and carbonyl (C=O), and the peaks at 1426 cm^−1^, 3377 cm^−1^, and 2902 cm^−1^ were assigned to ν(C-O), ν(OH), and ν(CH_2_), respectively. All the FT-IR spectra bands of five TOC samples were almost the same. It indicates that the introduction of various metal ions into cellulose-COONa through ion exchange had no effect on the position and strength of these specific peaks.

### 2.2. ^13^C-NMR Spectroscopy Analysis

**Figure 4:**
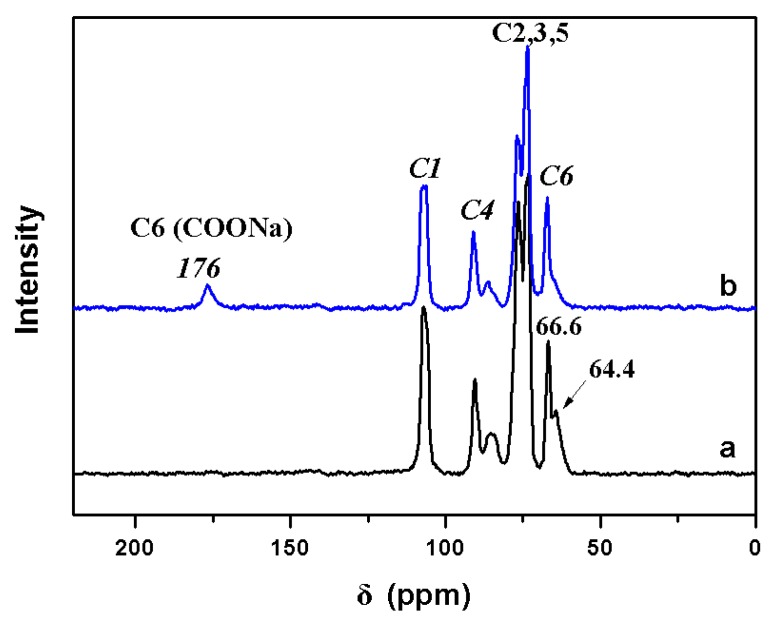
Solid-state ^13^C-NMR spectra of the original cellulose (**a**) and cell-COONa (**b**).

Solid-state ^13^C-NMR spectra of original cellulose and TEMPO-oxidized cellulose sample are shown in Figure 4. For the original cellulose, the typical signals of cellulose appear at 107 ppm (C1), 90 and 85 ppm (C4), 76 and 73 ppm (C2, C3, and C5), and 66.6 and 64.4 ppm (C6). A new peak at 176 ppm (see Figure 4b) is ascribed to the sodium carboxylate carbons in the oxidized cellulose, when compared with the original cellulose (see Figure 4a). These results are consistent with the previous relevant reports [16,17].

From Figure 4, we find that cellulose-COONa had nearly no influences on the chemical shift and the pattern of C1 or C4 in solid-state ^13^C-NMR spectra. However, the oxidized cellulose has large resonance peaks due to the original C6 primary alcohol groups of the glucose at about 67.3 ppm. Meanwhile, the signal at 64.4 ppm for noncrystalline C6 carbons sharply decreases and disappears when it is compared with the original cellulose, which indicates a selective oxidation of the primary OH-units [18]. Thus, it is possible to conclude that selective oxidation occurred at the C6 primary alcohol groups of wood pulp in TEMPO-NaBr-NaClO system.

### 2.3. TG Analysis

**Figure 5:**
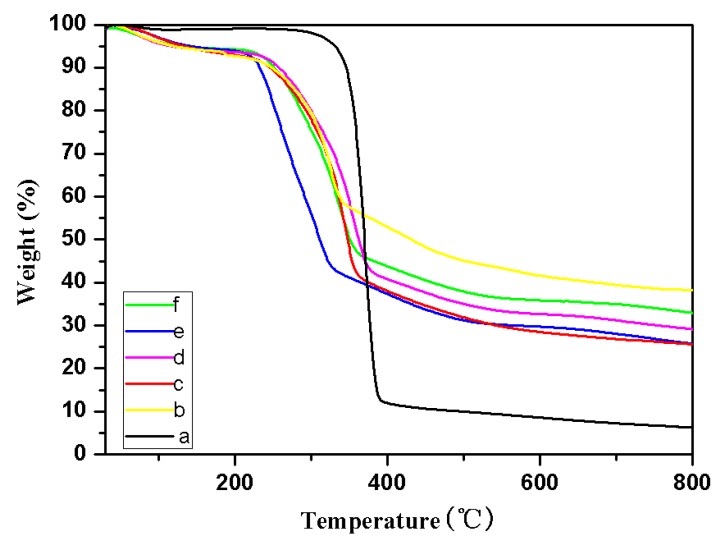
TG curves of cellulose (a), cellulose-COOZn (b), cellulose-COOMg (c), cellulose-COOCa (d), cellulose-COONa (e), and cellulose-COOBa (f) under N_2_.

**Figure 6:**
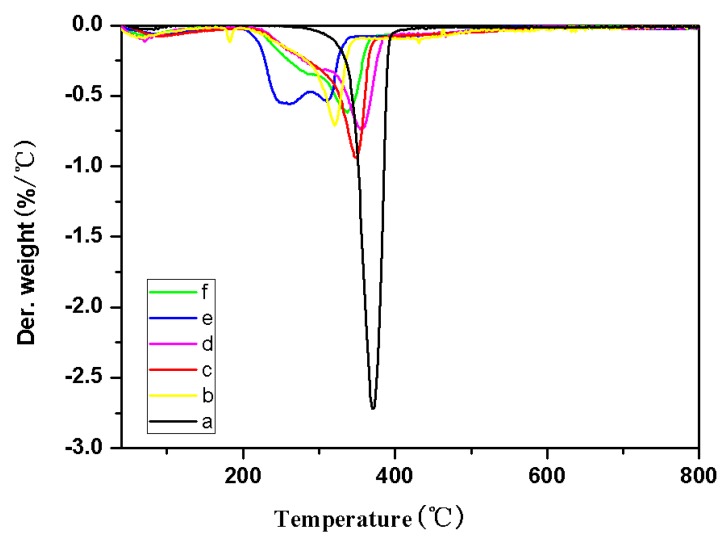
DTG curves of cellulose (a), cellulose-COOZn (b), cellulose-COOMg (c), cellulose-COOCa (d), cellulose-COONa (e), and cellulose-COOBa (f) under N_2_.

The thermal stability of cellulose and TOC were studied by thermal weight loss under nitrogen atmosphere. The TG and DTG curves were showed in Figure 5 and Figure 6, respectively. The TG and DTG data of the cellulose and TOC were listed in Table 1 in detail.

As can be seen from Figure 5 and Figure 6, and Table 1, the initial decomposition temperature (T_0.1_), maximum mass loss temperature (T_max_) and peak temperatures of derivative TG (DTG_peak_) of all TOC are lower than that of the pristine cellulose. It indicates that thermal stability of the original cellulose was significantly reduced after modification. It is possibly because of the increase of carboxyl groups and the addition of metal ions due to the decrease in crystallinity of TOC during the oxidation and ion-exchange [19].

However, the maximum weight loss rate (MMLR) of TOC was significantly lower than that of cellulose from Table 1. Some possible reasons can be speculated in that various TOC which contain more carboxyl groups will release a large amount of nonflammable gas (CO_2_) and dilute the flammable gas during degradation at a lower temperature. Additionally, it is more easily to generate carbon residue at a relatively low temperature [20].

We also found that the amount of residual combustion residue of TOC was significantly increased from Table 1. Generally, the formation of combustion residue will block oxygen ingress, slow down heat transfer, and prevent flame spread, and the more residual combustion residue, the more obvious are these effects during the process of polymer degradation.

### 2.4. MCC Analysis

**Figure 7:**
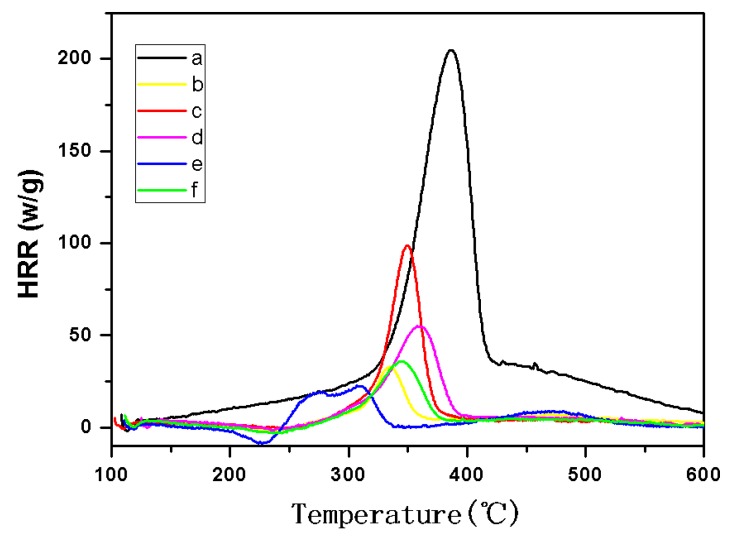
The heart release rate (HRR) curves of cellulose (**a**), cellulose-COOZn (**b**), cellulose-COOMg (**c**), cellulose-COOCa (**d**), cellulose-COONa (**e**), and cellulose-COOBa (**f**) at 1 K/s heating rate.

**Figure 8:**
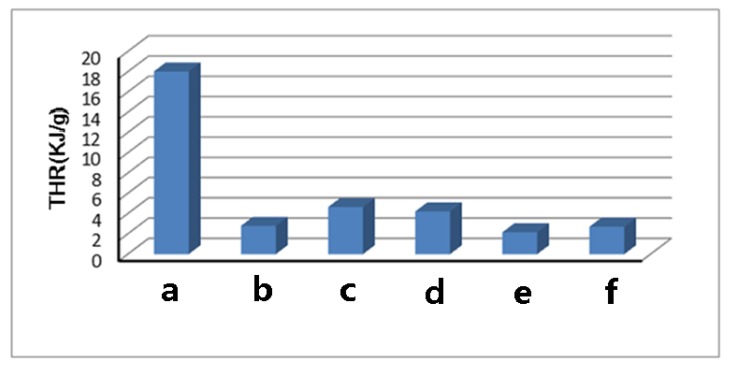
The total heart release (THR) of cellulose (a), cellulose-COOZn (b), cellulose-COOMg (c), cellulose-COOCa (d), cellulose-COONa (e), and cellulose-COOBa (f).

The flame retardancy of cellulose and TOC were tested and characterized using microscale combustion calorimetry (MCC). MCC analysis is a new, rapid, and convenient method that has become an efficient tool for characterizing newly synthesized flame-resistant polymer materials in recent years [21,22,23]. The heat release rate (HRR) curves for cellulose and its TOC were shown in Figure 7. The total heat release (THR) curves for cellulose and its TOC were shown in Figure 8. The other MCC data were shown in Table 2.

From Figure 7, the peak heat release rate (pHHR) of TOC was significantly reduced compared to that for pure cellulose. The pHHR of cellulose-COONa was reduced by nearly 90%, when compared with the original cellulose, in which the effect was significant. Simultaneously, the THR of cellulose is 18 KJ/g, and the THR of all TOC is also significantly reduced (Table 2, Figure 8). It is also seen that the amount of combustion residue (Table 2) of the TOC is increased, which is consistent with the results of TG analysis.

Other studies have shown that the presence of metal oxides in polymers can promote the formation of carbon residue [24,25]. Therefore, we can infer that, owing to the presence of a large amount of metal ions in TOC, the amount of combustion residue increased, thereby further reducing the THR.

### 2.5. LOI Test Analysis

The LOI test is one of the most commonly used test methods in studying the combustion properties of materials. The larger the value of LOI, the higher the oxygen concentration required for the material to burn, the harder it is to burn the material, and the better its flame-retardant performance. The LOI test was conducted to investigate the flame resistance of wood pulp before and after the modification. The corresponding results from the test are summarized in Table 3.

The LOI value of the original cellulose is 19%, and the LOI values of all TOCs products are more than 25%, wherein the highest LOI value is that of cellulose-COOZn is 34%. As can be seen from the results, the introduction of carboxyl groups and metal ions by selective oxidation of cellulose, the flame retardancy of the material is improved to some extent.

## 3. Materials and Methods

### 3.1. Materials

Sodium bromide (NaBr) (Guangdong Guanghua Chemical Factory Co., Ltd., China), 2,2,6,6-tetramethylpiperidine-1-oxyl radical (TEMPO, 98%), (J&K Scientific Ltd, Shanghai, China), and ten mass % (10 wt%) sodium hypochlorite (NaClO) solution (Tianjin Dingshengxin Chemical Industry Co., Ltd., China). Other chemicals were analytical grades and were used without prior purification. Cellulose wood pulp (Shandong Sun Paper Industry Joint Stock Co., Ltd., Jining, China) was used as the original cellulose sample.

### 3.2. Synthesis of TOC

The wood pulp (16 g) was suspended in purified water (1200 mL) which contained TEMPO (0.25 g, 0.1 mmol/g cellulose) and sodium bromide (1.65 g, 1.0 mmol/g cellulose). Then, a predetermined amount of the 10% NaClO solution (78 mL, 5.0–10.0 mmol/g cellulose) was added to the slurry in dropwise, and the mixture was mechanically stirred at room temperature. Meanwhile the pH of the mixture system was maintained to be 10.5 until no NaOH (0.5 mol/L) consumption was observed in the reaction process. The oxidation was quenched by adding ethanol (ca.10 mL). The oxidized cellulose was washed thoroughly with purified water and then ethanol by filtration. The wet product was dried by lyophilization, followed by vacuum-drying at 50 °C for about 48 h, and finally weighed to measure the mass recovery ratios.

After being TEMPO-oxidized, the wet product was suspended in an aqueous metal salt solution (1200 mL). The molar concentration of metal salt dissolved in the aqueous cellulose slurry was set to be about three times as much as original wood pulp in the slurry. After stirring the cellulose slurry at room temperature for 6 h, the fibrous cellulose fraction was washed thoroughly with a great deal of deionized water (ca. 4000 mL) followed by filtration. Then the filter cake was dried by lyophilization followed by vacuum drying at 50 °C for 48 h. The following metal salts (magnesium chloride, calcium chloride, zinc chloride, and barium chloride) were used for the ion-exchange treatments. The resulting metal-exchanged TOC was labeled as Cellulose-COOM, where M = Na, Mg, Ca, Zn, or Ba.

### 3.3. Characterization of TOC

#### 3.3.1. Fourier Transform Infrared (FT-IR) Spectroscopy

The structural changes were measured with a FT-IR NICOLET5700 spectrometer (Thermo Nicolet Corporation, Verona, State abbreviation, USA). Samples were studied as KBr pellets (1% in anhydrous KBr). Spectra were recorded in the range of from 4000 to 400 cm^−1^ with a resolution of 2 cm^−1^, and 20 scans were accumulated. In order to ensure the accuracy of detection, a blank run was made prior to every sample, and the spectrum of the blank was subtracted from the spectrum of the sample.

#### 3.3.2. ^13^C-NMR Spectroscopy 

In order to further analyze the structure of before and after oxidized cellulose, a solid-state ^13^C-NMR was used. The spectra were recorded on a Bruker AVANCE_III 300 Superconducting solid spectrometer (Brugg Group, Bruker, Swizterland) with operating frequency of 75.5 MHz and the main magnetic field strength of 7.05 T, CP/TOSS pulse sequence, 75,000 Hz spectral width, 4096 data points, 5 μs pulse length, and 5 s relaxation delay. Scans of up to 1024 were accumulated.

#### 3.3.3. Thermogravimetry (TG) 

The thermal stabilities of the samples were carried out by recording TG curve on a thermogravimetric analysis equipment (Q500, TA Instrument, Newcastle, USA). Approximately 8 mg of each sample was heated to 900 °C at a heating rate of 10 °C/min. All of the measurements were performed under a nitrogen atmosphere with a gas flow of 20 mL/min in order to prevent any thermo-oxidative degradation. TG measurements were duplicated in order to assess their reproducibility.

#### 3.3.4. Microscale Combustion Calorimetry (MCC)

The samples were pyrolyzed in a microscale combustion calorimetry (Govmark MCC-2, Govmark Organization, Inc.NewYork, USA) to 900 °C at 1 k/s under nitrogen atmosphere. The volatiles were swept out continuously by a N_2_ flow (80 mL/min), mixed with a metered flow of oxygen gas (20 mL/min), and completely combusted at 900 °C. Consumption rate of O_2_ was measured continuously. The heat release results were taken as the average of five measurements for each sample.

#### 3.3.5. Limiting Oxygen Index (LOI) Test

The flame retardancy of the prepared samples was tested by the HC-2 type limiting oxygen index meter (Nanjing Jiangning District Analytical Instrument Factory, China) according to GB4545-1997. Each fibrous sample was directly compressed, and the sample size was 100 mm × 37 mm × 2 mm (ca. 3 g).

## 4. Conclusions

The C6 primary hydroxyl group of cellulose can be electively oxidized to a carboxyl group by TEMPO-mediated oxidation system, and further various metal ions are introduced in the polymers by ion-exchange method. The thermal stabilities of the prepared TOC were significantly reduced after modification because of the increase of carboxyl groups and the addition of metal ions during the oxidation and ion- exchange due to the decrease in crystallinity of TOC. However, the MMLR of TOC was significantly lower than that of cellulose. This can be speculated in that various TOC which contain more carboxyl groups will release a large amount of nonflammable gas (CO_2_) and dilute the flammable gas during degradation at a lower temperature. Additionally, it is more easily to generate combustion residue at a relatively low temperature. Furthermore, it can be seen from the data of MCC that the HRR and THR of TOC were significantly reduced. The LOI values of all TOCs products were much higher than 25%, which indicated that the flame retardancy of the modified cellulose was improved.

## Figures and Tables

**Table 1:** The TG and DTG data of cellulose and TEMPO-oxidized cellulose (TOC).

Sample	T_0.1_ (°C)	DTG_peak_ (°C)	T_max_ (°C)	Combustion Residue (800 °C, wt%)	MMLR (%/°C)
cellulose	300	367	367	6.20	2.82
cellulose-COOZn	231	319	319	33.05	0.73
cellulose-COOMg	225	339	339	25.64	1.14
cellulose-COOCa	240	349	349	29.08	0.77
cellulose-COONa	229	260;307	260	25.70	0.56
cellulose-COOBa	243	334	334	32.92	0.61

T_0.1_: Temperature in weight loss of 10%; DTG_peak_: Peak temperatures of derivative TG; T_max_(°C): Maximum mass loss temperature; MMLR: Maximum mass loss rate.

**Table 2:** Microscale combustion calorimetry (MCC) data of cellulose and TOC.

Samples	pHHR (w/g)	T_pHHR_ (°C)	THR (KJ/g)	Combustion Residue (600 °C, %)
cellulose	204.7	388	18.0	8.57
cellulose-COOZn	33.0	335	2.8	35.51
cellulose-COOMg	98.8	350	4.7	28.55
cellulose-COOCa	55.0	358	4.2	32.65
cellulose-COONa	22.7	307	2.2	29.73
cellulose-COOBa	36.0	345	2.7	35.89

**Table 3:** The result of LOI of cellulose and TOC.

Samples	LOI (%)
Cellulose	19
cellulose-COOZn	34
cellulose-COOMg	27
cellulose-COOCa	25
cellulose-COONa	27
cellulose-COOBa	25
